# Cannabidiol reduces lipopolysaccharide-induced vascular changes and inflammation in the mouse brain: an intravital microscopy study

**DOI:** 10.1186/1742-2094-8-5

**Published:** 2011-01-18

**Authors:** Lourdes Ruiz-Valdepeñas, José A Martínez-Orgado, Cristina Benito, África Millán, Rosa M Tolón, Julián Romero

**Affiliations:** 1 Laboratorio de Apoyo a la Investigación, Hospital Universitario Fundación Alcorcón and Centro de Investigación Biomédica en Red sobre Enfermedades Neurodegenerativas (CIBERNED), Alcorcón, 28922, Madrid, Spain; 2 Neonatología, Servicio de Pediatría, Hospital Universitario Puerta de Hierro, Majadahonda, 28222, Madrid, Spain; 3 Dept. of Biochemistry, Francisco de Vitoria University, Pozuelo de Alarcón, 28223, Madrid, Spain

## Abstract

**Background:**

The phytocannabinoid cannabidiol (CBD) exhibits antioxidant and antiinflammatory properties. The present study was designed to explore its effects in a mouse model of sepsis-related encephalitis by intravenous administration of lipopolysaccharide (LPS).

**Methods:**

Vascular responses of pial vessels were analyzed by intravital microscopy and inflammatory parameters measured by qRT-PCR.

**Results:**

CBD prevented LPS-induced arteriolar and venular vasodilation as well as leukocyte margination. In addition, CBD abolished LPS-induced increases in tumor necrosis factor-alpha and cyclooxygenase-2 expression as measured by quantitative real time PCR. The expression of the inducible-nitric oxide synthase was also reduced by CBD. Finally, preservation of Blood Brain Barrier integrity was also associated to the treatment with CBD.

**Conclusions:**

These data highlight the antiinflammatory and vascular-stabilizing effects of CBD in endotoxic shock and suggest a possible beneficial effect of this natural cannabinoid.

## Background

Endotoxic shock (ES) is a life-threatening condition with mortality rates of 40-70% that usually takes place in seriously ill, immunologically compromised patients [1]. In ES, usually secondary to Gram-negative bacterial infection, there is a severe impairment of vascular, coagulant, immune and inflammatory responses of the host [2]. The lipopolysaccharide (LPS), a component of the cell wall of gram-negative bacteria, mediates many of the alterations leading to ES. LPS profoundly impairs endothelial functions, promoting intravascular coagulation, disruption of the endothelial wall and intense vasodilation and hypotension. The therapeutic usefulness of potent antiinflammatory agents as steroids remains controversial [2]. Thus, the search for effective treatments in ES is still demanding.

Encephalopathy is a common complication in ES patients, usually appearing very early in the pathologic process and determining the prognosis [3]. LPS injection, by inducing both endothelial and astrocytic cell dysfunction [4,5], is particularly harmful for brain circulation, impairing cerebrovascular autoregulation [3,6]. Autoregulatory responses of brain arteries and arterioles guarantee a constant cerebral perfusion during systemic blood pressure changes, being dependent on a normal endothelial function, in particular during hypotension [7]. LPS also disrupts the coupling of local cerebral blood flow (CBF) with the activity of underlying neurons [3,4].

Cannabidiol (CBD] is a phytocannabinoid with well-known antiinflammatory and antioxidant properties [8,9]. El-Remessy et al [10] recently reported that CBD prevented inflammatory and oxidative damage and preserved endothelial integrity in an experimental model of diabetic retinopathy. Furthermore, CBD preserves cerebral circulation in pathological conditions such as brain ischemia [11]. Recent data support the clinical use of CBD for the treatment of a variety of damaging conditions, including nephropathy and diabetic cardiomyopathy. In particular, the antioxidant properties of CBD seem to play a major role in the protective effects of this phytocannabinoid against the oxidative and nitrosative stress induced by chemoterapy agents and by high glucose conditions [12,13].

In the present work, we aimed to test the beneficial effects of CBD in brain circulation and inflammation in an *in vivo *model of sepsis after parenteral injection of LPS. To that end, we opened a cranial window in adult mice to study vascular responses by intravital microscopy. Former studies using this method have demonstrated that topic application of LPS altered arteriolar responses [14]; however, few studies have so far reported on the effect of i.v. injection of LPS. Such a difference is relevant as LPS cannot readily cross the blood brain barrier (BBB) [15] and because, during the actual septic condition, both endotoxin release and leukocyte activation take place inside the intravascular space [16].

## Methods

### Mice and preparation for intravital microscopy

Adult C57BL/6J mice were maintained in a temperature-controlled specific pathogen-free facility with a strict 12-hour light/dark cycle and with free access to food and water. All experiments were performed in accordance to international and local guidelines as approved by an internal committee (86/609/EEC). Mice were anesthetized with ketamine plus medetomidine (50 mg/kg and 1 mg/kg, respectively). After removing the skin and muscle, a custom-made device was attached to the cranial surface and then fitted to the microscope. Body temperature was continuously monitored by a rectal probe and maintained constant with a thermal blanket. Blood pressure (BP) was monitored by a cuff tail device with a photoelectric sensor (NIPREM 645, Cibertec, Madrid, Spain). A cranial window (2 mm of diameter) was then opened with a high-speed drill, to gain direct access to the brain parenchyma. The tissue was kept humid constantly by subsequent additions of 200 μl-drops of 0.9% saline. Staining of superficial endothelium and microglia was performed by topical administration of *Griffonia simplicifolia *conjugated with fluorescein (Vector Laboratories, Burlingame, CA, USA), in a 0.9% NaCl solution, for 30 min. At the beginning of the experiment, at 90 and at 180 min, a 50 μL blood sample was obtained by tail puncture to determine blood gases (i-STAT, Abbot Laboratories, NJ, USA). At the end of the experiment mice were killed by decapitation and brains harvested, frozen and conserved at -80°C until use.

### Drug administration

To clearly observe the cerebrovascular tree throughout the entire experiment, 100 μl of a 70000 MW Texas red-conjugated dextrane solution (Invitrogen, Carlsbad, CA, USA) was administered through the tail vein. This approach stains blood plasma while leaving nucleated cells unstained [17]. Afterwards, vehicle (Tween/saline, N = 7), LPS (Sigma, St Louis, MO, USA; 1 mg/kg, N = 8), LPS+CBD (1 mg/kg + 3 mg/kg respectively; Tocris Bioscience, Bristol, UK, N = 7) or CBD alone (3 mg/kg, n = 5) were administered i.v. through the tail vein in a total volume of 100 μl. Doses of LPS and CBD were chosen based on previous data [5,18]. A single dose of CBD was chosen because its long half-life time [18] makes it appropriate for experiments lasting for 3 h as ours.

### Image acquisition and analysis

Observations were made using a Nikon 90i upright microscope coupled to a C1 scanhead confocal system with two laser sources (Arg 488 nm and He/Ne 543 nm). Once the area of interest was defined, 60 μm-thick stacks in the Z-axis (3 μm steps) were obtained with the Nikon EZ-C1 software, every 15 min for a total time of 180 min post drug administration. Three-dimensional constructs were analyzed and changes in the diameter of venules (internal diameter 39-112 μm) and third-order arterioles (internal diameter 14-50 μm) (at least 4 of each per animal) measured. Pial vessels of those diameters are considered as optimal for intravital studies on microvessel reactivity [19]. In addition, the total number of marginated cells (revealed as immobilized black dots inside the vessels) at each time point was counted and the accumulated amount was expressed per area unit (μm^2^). To that end, the total area corresponding to vessels was estimated in each field of observation by means of ImageJ (NIH) software.

### BBB integrity

70000 MW dextrane is unable to leave the blood vessels under normal conditions, but diffuses into the brain parenchyma when BBB integrity is compromised. In order to measure this phenomenon, laser-scanning micrographs were analyzed and fluorescence intensity across a cross-section of edematous vessels was measured. This allowed the analysis of fluorochrome distribution inside and outside the affected vessels as an index of BBB damage [20].

### Quantification of markers of oxidative stress

Concentrations of 4-hydroxynonenal (HNE) and of malondialdehyde (MDA) as markers of oxidative stress, were measured in frozen brain tissue by ELISA (OxiSelect HNE-His Adduct and OxiSelect MDA Adduct, Cell Biolabs, San Diego, CA, USA).

### COX-2, TNF-α and iNOS mRNA levels

mRNA levels of cyclooxygenase (COX)-2, tumor necrosis factor-alpha (TNF-α) and inducible nitric oxide synthase (iNOS) were quantified by qRT-PCR from frozen midbrains. Total RNA was extracted using the Tripure Isolation Reagent (Roche Diagnostics, Mannheim, Germany). Single-stranded complementary DNA (cDNA) was synthesized from 1 μg of total RNA using the Transcriptor First Strand cDNA Synthesis Kit (Roche Diagnostics, Mannheim, Germany). PCR primers and TaqMan probes were designed by Tib Molbiol (Berlin, Germany) as shown in table 1. For normalization, 18S primers and probe number 55 from Universal ProbeLibrary probes 1-165 (Roche) were utilized. Gene expression was quantified using the Quantimix Easy Probes kit (Biotools, Madrid, Spain) in concert with a LightCycler thermocycler (Roche). Standard curves were calculated for quantification purposes using ten-fold serial dilutions of cDNA from brain mouse. The transcript amounts were calculated using the second derivate maximum mode of the LC-sotfware version 4.0. The specific transcript quantities were normalized to the transcript amounts of the reference gene 18S. All further calculations and statistical analyses were carried out with these values referred to as relative expression ratios.

**Table 1:** Sequences for primers and probes employed in this study

*GENES*	*PRIMER/PROBE*	*SEQUENCES FOR PRIMERS AND PROBES*
**18S**	*sense*	AAATCAGTTATGGTTCCTTTGGTC
	*antisense*	GCTCTAGAATTACCACAGTTATCCAA
	***Probe #55***	Use universal ProbeLibrary, Roche Applied Science
**iNOS**	*sense*	GCTCCTCCCAGGACCACA
	*antisense*	GCTGGAAGCCACTGACACTT
	***Probe TaqMan***	6FAM-CACCTACCGCACCCGAGATGG--BBQ
**COX-2**	*sense*	TGACCCACTTCAAGGGAGTCT
	*antisense*	CTGTCAATCAAATATGATCTGGATGTC
	***Probe TaqMan***	6FAM-AACAACATCCCCTTCCTGCGAAGTT--BBQ
**TNF-α**	*sense*	GCCTATGTCTCAGCCTCTTCTCATT
	*antisense*	CCACTTGGTGGTTTGCTACGA
	***Probe TaqMan***	6FAM-CCATAGAACTGATGAGAGGGAGGCCATTT-BBQ

### Statistical analysis

Results are expressed as mean ± SEM of the indicated number of experiments. Changes in vessel diameter with time were compared between groups by 2-way ANOVA. Differences between groups in enzyme or protein expression were studied by 1-way ANOVA. Newman-Keuls post-hoc test was used for multiple comparisons. A p value of less than 0.05 was considered as statistically significant. Statistical analysis was performed using the 11.0.0 version of SPSS software (SPSS Inc.).

## Results

### Physiological data

Mice rectal temperature (37.5 ± 0.6, 37.7 ± 0.2 and 38.1 ± 0.4°C for VEH, LPS, and LPS+CBD, respectively, NS) measured in the first 30 min of the experiment indicated a mean decrease of 2°C, remaining stable then. There were no differences between groups throughout the experiment.

BP remained stable throughout the entire experiment and showed no differences between groups (mean BP [range]: 137 ± 14 [128-150], 141 ± 12 [123-154] and 149 ± 5 [130-155] mmHg for VEH, LPS and LPS+CBD, respectively, NS).

Blood gas values remained in the normal range until the end of the experiment, with no differences between groups (pH: 7.38 ± 0.01, 7.35 ± 0.02 and 7.39 ± 0.04; pO_2_: 47.7 ± 4.8, 45.6 ± 5.1 and 45.6 ± 2.7 mmHg; and pCO_2_: 66.3 ± 9.3, 71.4 ± 8.8 and 61.6 ± 6.3 mmHg, for VEH, LPS and LPS+CBD, respectively, NS).

### CBD counteracts LPS-induced vasodilation

**Figure 1:**
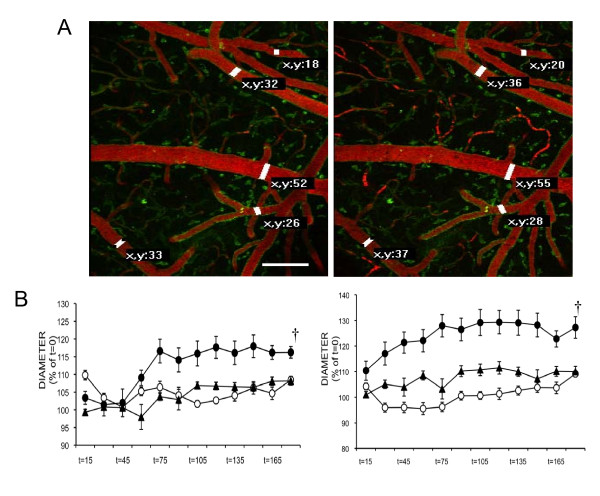
**CBD prevented LPS-induced vasodilation**. **(A) **Representative 60 μm Z-stacks of subpial vessels in the mouse brain reflect the increase in blood vessel diameter after LPS administration (t = 0, left; t = 120 min, right). X-Y labels represent diameter in micrometers. Vascular tree is evidenced by i.v. administration of 70000 MW Texas red-conjugated dextrane and endothelial and microglial cells were labelled with topical fluorescein-conjugated *Griffonia simplicifolia *(green). **(B) **Treatment with CBD (triangles) significantly decreased the LPS-induced changes (black circles) in venules (left) and arterioles (right), as compared to vehicle treated animals (open circles). Error bars represent mean ± SEM of 7-8 experiments. ^†^p < 0.05 by 2-way ANOVA. Scale bar: 250 μm.

LPS-induced sepsis-associated changes in cerebral blood flow are due to, among other factors, an excessive vasodilation [21]. As expected, LPS induced a sustained arteriolar vasodilation of up to 30 ± 3%, starting at t15 min and peaking at t75 min (Figure 1); LPS also induced a venular vasodilation of up to 15 ± 2% starting at t75. CBD blunted the vasodilator effect of LPS, so that in LPS+CBD arteriolar and venular dilation accounted only for 10 ± 2% and 5 ± 2%, respectively (2-way ANOVA p < 0.05 vs. LPS, F = 3.48 and F = 4.22 for venules and arterioles, respectively).

### CBD decreases LPS-induced cell margination

**Figure 2:**
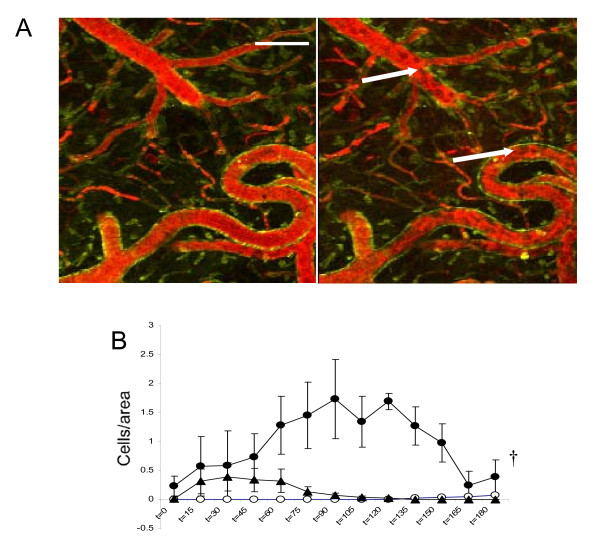
**CBD inhibited leukocyte margination**. **(A) **Representative 60 μm Z-stacks of subpial vessels at t = 0 (left) and t = 120 min (right) after LPS administration. Note the intense accumulation of nucleated cells in the luminal side of blood vessels at t = 120 min, detectable as static black dots (arrows). **(B) **Quantification of CBD effect on cell margination (expressed as immobilized cells/μm^2^), reflecting a dramatic inhibition of the process by this cannabinoid over the experimental time course. Vehicle (open circles), LPS (black circles) and LPS+CBD (triangles). Error bars represent mean ± SEM of 7-8 experiments. ^†^p < 0.05 by 2-way ANOVA. Scale bar: 250 μm.

To study leukocyte margination and diapedesis, of paramount importance in LPS-induced sepsis, the intravascular space was stained with 70000 MW Texas red-conjugated dextrane. Blood cells thus appear as "ghosts" inside the vessels [17]. With this approach, only cells that are stationary or dramatically slowed by adhesive interactions with the vessel wall can be detected [17,22]. The great majority of these unstained cells are leukocytes, as they exceed in high number other nucleated cell types [17]. Leukocyte margination was not observed in VEH at any time studied, whereas in LPS-treated animals the density of marginated leukocytes was significantly elevated. LPS+CBD blunted this effect (Figure 2) (2-way ANOVA p < 0.05, F = 1.87).

### LPS treatment compromised BBB integrity, and this effect was prevented by CBD

**Figure 3:**
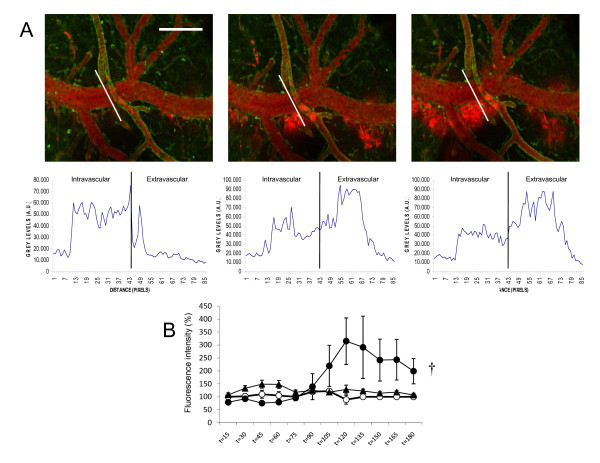
**CBD prevented LPS-induced extravasation, as an index of BBB integrity**. **(A) **Representative laser-scanning micrographs of microvasculature at t = 90 (left), t = 105 (center) and t = 120 min (right) after LPS administration. Note the increasing red signal outside the vessel, indicating extravasation of the high molecular weight, fluorescent dextrane, confirmed by the measurement of fluorescence intensity across a cross-section (white line) at each time point. **(B) **Representation of the variation with time of the extravasation as measured by fluorescence intensity in the extravascular portion, in LPS (black circles) and LPS+CBD (triangles) treated animals, as compared to vehicle (open circles). Error bars represent mean ± SEM of 7-8 experiments. ^†^p < 0.05 by 2-way ANOVA. Scale bar: 200 μm.

Mice receiving LPS showed a clear disruption of the BBB, as revealed by extravasation of the fluorescently-labelled dextrane starting 45 min after administration (Figure 3). This phenomenon has been previously employed for the quantification of BBB integrity [20]. In animals showing BBB alterations, CBD treatment dramatically reduced the extent of dextrane extravasation (p < 0.05 by 2-way ANOVA, F = 2.23) (Figure 3). Interestingly, no vehicle-treated animals showed significant changes in BBB integrity.

### CBD does not modify HNE or MDA concentrations

No differences were observed between groups in the concentration of HNE (1.37 ± 0.4, 1.15 ± 0.3 and 1.39 ± 0.5 μg/ml, for VEH, LPS and LPS+CBD, respectively, NS) or MDA (3.2 ± 0.3, 2.9 ± 0.4 and 3.3 ± 0.5 μg/ml, for VEH, LPS and LPS+CBD, respectively, NS) in brain tissue.

### CBD reduces LPS-induced expression of COX-2, TNF-α, and iNOS

**Figure 4:**
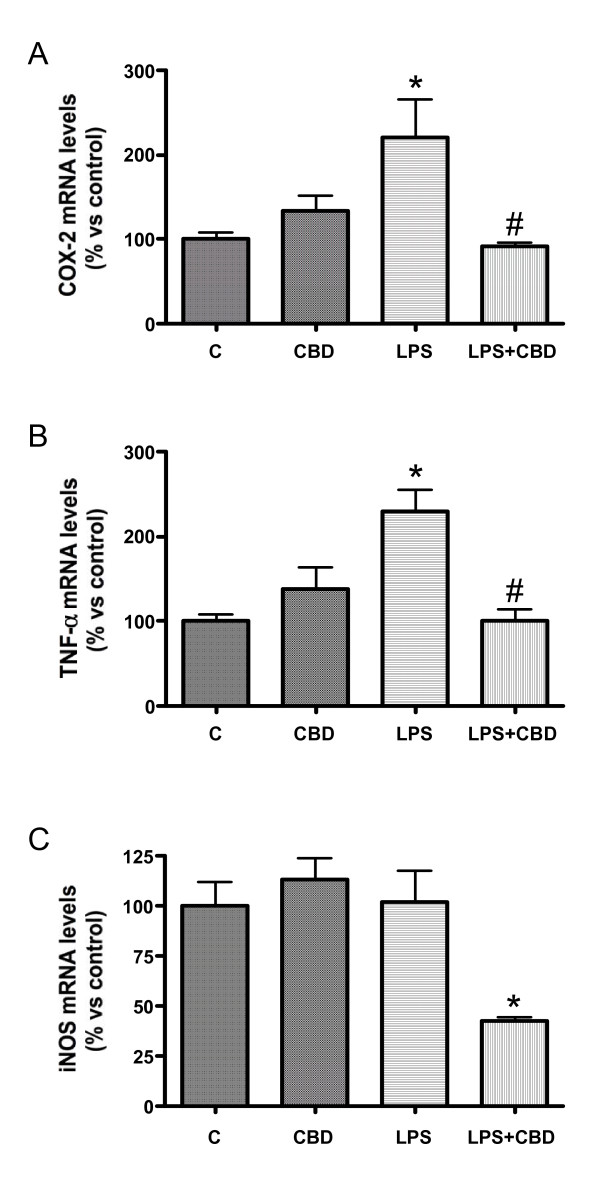
**CBD treatment modifies the pattern of induction of COX-2 (A), TNF-α (B) and iNOS (C) mRNAs, as measured by quantitative real time PCR**. Error bars represent mean ± SEM of 7-8 experiments. *p < 0.05 vs control. #p < 0.05 vs LPS by ANOVA.

LPS triggers a massive inflammatory response involving cellular mediators such as cytokines and prostaglandins [21]. Thus, we aimed to quantify the expression of one crucial cytokine (TNF-α) and of some key enzymes (COX-2 and iNOS). Taken together, our results confirm the development of a proinflammatory environment in LPS-treated mice brain, with increases in mRNA levels for TNF-α and COX-2 (Figure 4). iNOS expression was not modified in LPS vs vehicles although CBD-treated mice exhibited significantly lower expression of this enzyme. LPS-induced increases of TNF-α and COX-2 were dramatically reduced by CBD (Figure 4).

No differences between VEH and animals treated with CBD alone were observed in any of the aforementioned determinations.

## Discussion

CBD is a natural cannabinoid lacking psychotropic effects. This fact, together with its well-known antiinflammatory, antioxidant and neuroprotective effects, has focused research on its possible therapeutic relevance [8,23]. We here report that CBD counteracts some of the inflammatory responses associated to LPS in the mouse brain.

In the present work we observed that parenterally-introduced LPS induced dramatic arteriolar dilation, starting as early as 30 min after i.v. injection. Previous *in vivo *experiments on the effect of parenteral or intracerebral LPS administration (1 mg/kg or more) on brain circulation in rodents measured local or global CBF [3,6,15]. In those studies, brain hyperemia took place 1 h after LPS administration. We did not directly measure CBF, but the sequential and parallel increase in venular diameter after LPS administration likely reflected an increase in CBF as, due to the poor reactivity of the cerebral venous myocytes, brain venule diameter is mostly dependent on CBF [19]. Brain hyperemia after abnormal brain vasodilation represents a lack of autoregulation, due to endothelial and glial dysfunction [3]. Administration of CBD blunted the arteriolar dilation, so arteriolar diameter remained similar to control throughout the experiment. Besides, venular diameter after CBD administration remained similar to control. Altogether, these results suggest that in the presence of CBD, CBF remained similar to control.

On the other hand, the compromise of BBB integrity is a common feature of LPS-associated encephalitis and may be caused by the disruption of endothelial tight junctions mainly by the action of several cytokines. Increased BBB permeability leads to secondary lesions that worsen with increased duration of septic shock and correlate with poor outcome [16]. CBD reduced BBB alteration, in agreement with a recent report showing that CBD prevents endothelial cell inflammatory responses and preserves barrier functions in a murine model of experimental diabetes [24]. Furthermore, our data showing that CBD was able to prevent cellular margination match with recent observations *in vivo *in which CBD decreased the expression of adhesion molecules as well as chemotaxis in experimental models of inflammation and tissue injury [12,13].

LPS is known to induce COX-2 in neurons and glial cells, subsequently increasing COX metabolites of arachidonic acid, which contributes to LPS-induced cerebral hyperemia [15]. At high concentrations, CBD inhibits COX-2 activity [18], an effect dependent on the cell type, as it is not observed in tumoral cells [25]. Our results confirm previous observations by Costa et al [26] showing that oral administration of CBD diminishes carrageenan-induced paw inflammation in rat by decreasing COX activity and edema formation.

Since hemodynamic changes in ES are triggered by the massive inflammatory reaction induced by LPS as well as by the increased oxidative stress, CBD beneficial effects could also derive both from its antiinflammatory and antioxidant properties [27]. Binding of LPS to specific receptors in brain endothelial cells triggers a series of signaling events leading to the increase of cytokine production [28]. These cytokines participate in the disruption of BBB integrity and induce brain vessel dilation [21,29]. TNF-α is a major mediator in septic encephalopathy, as mice deficient in TNF-receptor 1 are more resistant to LPS-induced changes [30]. CBD exerts a potent immunosuppressive effect *in vivo*, reducing production of TNF-α and other cytokines from immune cells [8,12,13,18].

LPS is one the most important stimuli for the induction of iNOS in brain cells [15]. iNOS induction leads to massive NO production, inducing endothelial cytotoxicity by direct damage and by increasing oxidative stress and inflammation [31], and impairing cerebrovascular autoregulation [3]. However, due to the limited ability of LPS to cross the BBB, the effect of parenteral LPS on brain iNOS mRNA levels could not be observed before 6 h after injection [5]. In agreement, we did not find an increase of iNOS expression in brain during our 3 h period of study after LPS injection. Thus, the CBD-induced decrease of iNOS expression likely corresponded with the prevention by CBD of iNOS induction in brain due to the experimental procedure. A similar effect on iNOS induction has been described for CBD newborn mice brains after manipulation to perform oxygen-glucose deprivation of forebrain slices [32]. CBD is known to prevent iNOS expression through inhibition of MAPK and NF-κB signaling [12,13,33,34], an observation that may be especially relevant in advanced stages of circulatory shock, when iNOS contribution to NO production seems to be maximal [35]. Remarkably, peroxynitrite formation (known to participate in pathophysiological alterations of shock) has been found to follow a similar time course to iNOS expression after challenge with LPS in rats (reviewed in 35).

We did not observe any difference between groups in brain concentration of oxidative stress markers as HNE or MDA. LPS administration leads to a brief transient increase of oxidative stress markers in brain, observed shortly after injection [36]. Nevertheless, the sustained and significant increase of these markers is observed 6 h after LPS administration, thus beyond our experimental period, and lasts for at least 24 h, being mainly due to cytokine-induced activation of microglial cells [36].

Finally, the complex pharmacological profile of CBD may explain some of our data [reviewed in [37]]. Thus, and although the possible mediation of cannabinoid receptors has not been analyzed in the present experiments, it is important to note that recent reports suggest that CBD effects on LPS-induced inflammation are receptor-independent [34]. However, CBD antagonizing properties on CB_1 _receptors might underlie some of the observed effects, as CB_1 _receptor blockade prevents the primary hypotensive response to LPS [27]. Furthermore, CB_1 _receptor blockade has been proposed to improve survival in ES [38]. In addition, CBD might also partially activate CB_2 _receptors, which play a crucial role in the regulation of the immune response against sepsis in an animal model of cecal ligation and puncture [39]. Furthermore, its activity as a CB_2 _receptor inverse agonist could partially account for these actions since CB_2 _receptor inverse agonism reduces clinical signs of inflammation and cell migration [40]. Finally, CBD may also alter inflammatory processes by targeting the abnormal CBD receptor [41], as this receptor partially mediates the hypotensive effects of anandamide and other cannabinoids [42,43].

## Conclusions

In conclusion, CBD blunted LPS-induced changes in vessel diameter and permeability as well as leukocyte margination, effects that were associated with modulation of cytokine and NO production. However, more studies on the optimal dosage regime, timing of effectiveness and response in other models of sepsis are warranted before considering CBD as a candidate for treatment in humans.

## Competing interests

The authors declare that they have no competing interests.

## Authors' contributions

LR-V carried out intravital microscopy experiments and quantification of images and of expression of inflammatory parameters; JAM-O. designed the experiments and statistical anaylisis and wrote the manuscript; CB quantified the expression of inflammatory parameters; AM carried out intravital microscopy experiments; RMT quantified the expression of inflammatory parameters; JR designed the experiments, carried out intravital microscopy experiments and quantification of images and wrote the manuscript. All authors have read and approved the final version of this manuscript.
